# Visceral leishmaniasis in Northeast Brazil: What is the impact of HIV on this protozoan infection?

**DOI:** 10.1371/journal.pone.0225875

**Published:** 2019-12-05

**Authors:** Uiara Regina Silva de Lima, Luciano Vanolli, Elizabeth Coelho Moraes, Jorim Severino Ithamar, Conceição de Maria Pedrozo e Silva de Azevedo

**Affiliations:** 1 Post-graduation Program of Health Science, Federal University of Maranhão, São Luís, MA, Brazil; 2 Medicine Graduate Program, Federal University of Maranhão, São Luís, MA, Brazil; 3 President Vargas Hospital, São Luís, MA, Brazil; 4 Department of Medicine, Federal University of Maranhão, São Luís, MA, Brazil; Imperial College London, UNITED KINGDOM

## Abstract

**Background:**

The aim of this study was to compare cases of Visceral Leishmaniasis (VL) with and without HIV in a state in northeastern Brazil.

**Methodology:**

We performed a comparative study in the state’s referral hospital for infectious/parasitic diseases located in Northeast Brazil between January 2007 and July 2017. The data obtained using this protocol were analyzed with SPSS.

**Principal findings:**

In total, 252 patients were evaluated, including 126 with coincident VL/HIV and 126 with VL alone. Both groups primarily consisted of male patients. The most commonly affected ages were 30–39 years in the coinfected group and 19–29 years in the VL group (*p* < 0.001). Fever and anorexia (*p* = 0.001), which were more common in those with VL alone, were frequently observed, while diarrhea, vomiting, bleeding and dyspnea were more common in patients with VL/HIV coinfection (p<0.005). According to the hemogram results, leukocyte levels were lower in the VL group (*p* < 0.0001). Additionally, AST (aspartate aminotransferase) and ALT (alanine aminotransferase) levels differed between the groups, with higher levels in patients with VL (*p* < 0.001). On average, HIV was diagnosed 2.6 years before VL (*p* < 0.001), and VL relapse was observed only in the coinfection group (36.5% of cases). Fever (*β* = +0.17; *p* = 0.032) in the first VL/HIV episode was identified as a risk factor for relapse (*R*^2^ = 0.18). More deaths occurred in the VL/HIV group (11.1%) than in the VL group (2.4%).

**Conclusion/Significance:**

VL/HIV was found to be prevalent among young adults, although the median patient age was higher in the VL/HIV group. The classic symptomatology of VL was more common in patients not coinfected with HIV; therefore, attention is needed in patients with HIV who present with any symptoms that suggest the presence of VL, especially in endemic areas. No cases of VL relapse occurred in patients without HIV, and death was more common in the VL/HIV co-infected group.

## Introduction

Visceral leishmaniasis is an infectious parasitic disease transmitted by *Lutzomyia longipalpis*, and *Leishmania infantum chagasi* is the main causative agent in South America [1]. VL has a large impact on Latin America, with 55,530 human cases reported between 2001 and 2016. In Brazil, 41,263 cases were reported during a recent 10-year period (2007–2017), with the northeast region accounting for 49.9% [2]. Since the first case of VL/HIV coinfection was reported in 1985, increasing attention has been paid in Mediterranean and northern European countries regarding the likelihood of this association affecting the severity of both diseases [3]. By 2007, 35 countries had already reported cases of VL/HIV coinfection [4]. In Brazil, coinfection was first reported in 1990 in a patient from the Northeast region [5]. In the 2007–2017 period, data from Notification of Injury Information System-Sinan Net showed notifications of 3,037 cases of VL/HIV coinfection, with a rate of 7.36% among cases of leishmaniasis. By comparison, 982,129 cases of human immunodeficiency virus/acquired immune deficiency syndrome (HIV/AIDS) were registered through June 2018 [6]. In recent years, this detection rate has been declining in Brazil, with a reduction of 9.4% between 2007 and 2017, whereas the northeastern region has shown an increasing trend (24.1%) of AIDS detection [7]. This explains its association with other endemic diseases, such as VL, which has also increased in frequency in urban locations, thereby accelerating the clinical evolution of both HIV and VL due to cumulative immunosuppression [8,9]. Due to altered epidemiological profiles of both HIV and VL, the risk of exposure to the diseases has grown, with the risk of non-HIV-infected individuals developing VL increasing by 100–2320-fold in endemic areas. As the clinical features of VL may evolve with severity, early diagnosis is needed to avoid further complications [10]. Moreover, coinfected patients have reduced responses to therapy, resulting in increased risks of recurrence and mortality [11]. Treatment of VL in Brazil is limited to the use of antimonials, amphotericin B and pentamidine. Following the Ministry of Health recommendations in 2013, liposomal amphotericin B has been the drug of choice for VL/HIV coinfection, initially with a total dose from 20 to 24 mg/kg [12]. Presenting a new recommendation in 2015 with a total dose of 24 to 40 mg/kg of liposomal amphotericin B [10], this drug of choice is also indicated for signs of severe VL, in addition to coinfection with HIV, pregnancy, and very young or old age (< 2 years, > 65 years).

Overall, there are high incidence rates of VL in Maranhão: over 10 years (2007–2017) in this state, 11.9% and 11% of Brazilian cases of leishmaniasis and VL/HIV coinfection were reported, respectively. Indeed, Maranhão ranked second in the country in the number of cases during this period [2]. In an effort to improve knowledge of this pathology, especially regarding epidemiological, clinical, and laboratory data related to VL/HIV coinfection, this study sought to identify particularities in the clinical and laboratory presentation of VL, irrespective of its coinfection with HIV. This information may contribute to improving the diagnosis and therapeutic management of patients with VL/HIV coinfection.

## Methods

### Type of study, location, and population

This comparative study of adults at least 18 years old was conducted between January 2007 and July 2017 and divided into two steps: (i) a retrospective study of patients diagnosed with VL/HIV coinfection between 2007 and 2015 and (ii) a prospective study of coinfected patients diagnosed between 2016 and 2017. Data were also collected retrospectively from the records of VL patients without HIV coinfection who were treated at the hospital during the study period. We used a protocol sheet adapted from the monitoring and control handbook for VL from the Health Ministry, which was named the investigation sheet of death from VL [13], to record the following variables: sociodemographic (sex, age, occupation, place of residence), epidemiological (place of infection, other places of residence in the last 12 months), background (other diseases and treatments), and physical evaluation variables (hydration level, abdominal protrusion, hepatosplenomegaly, swelling); HIV and leishmaniasis treatment; laboratory examinations (complete blood count, AST, ALT, urea, creatinine, glycemia, CD4 T-lymphocyte count, viral load, bone marrow test); and disease evolution (recurrence, death, hospital release). A case was considered new when the patient had no history or previous record of VL; otherwise, a case was deemed recurrent. The study was conducted at Maranhão’s referral hospital for infectious parasitic diseases, which serves the adult population, including approximately 4640 patients with HIV/AIDS, under active monitoring.

### Inclusion criteria

The retrospective study included patients with records of VL irrespective of HIV coinfection who had complete epidemiological, clinical, and laboratory data. The prospective study included patients with positive serology for HIV and a parasitological diagnosis of VL who were receiving treatment for coinfection at the referral hospital. The VL group consisted of patients with a parasitological diagnosis of VL and negative serology for HIV who were treated at the referral hospital. All patients included in the prospective study provided written informed consent for participation.

### Diagnosis

#### Visceral leishmaniasis

Suspected case: Patients with fever (persistent and long-lasting) and splenomegaly [2].

Confirmed case: Suspected clinical cases with at least one of the following:

Parasitological—VL indicated by the presence of the causative parasite in the bone marrow (amastigotes found in smears from bone marrow aspirate stained with Giemsa) [2]. Serological—VL indicated by results of the indirect fluorescent antibody test that is widely used for diagnosing VL and is currently provided by Brazil’s Unified Health System [2]. Another method used was Rapid Immunochromatographic Test Diagnosis Human Leishmaniasis.

#### Visceral leishmaniasis with HIV

Suspected case: Hepatomegaly or splenomegaly associated with fever (persistent and long-lasting) and cytopenia [14].

Confirmed case: Suspected clinical signs with parasitological diagnosis, i.e., amastigotes found in smears from bone marrow aspirate stained with Giemsa [2].

#### HIV

HIV was diagnosed using ELISA and the HIV rapid test, which are routinely performed at the referral state hospital following recommendations from the Health Ministry. Other laboratory data used as references were obtained using the automated system CELL DYN 4000 provided by CEDRO, São Luís, MA, 2002.

### Treatment

#### Visceral leishmaniasis

As recommended by the Ministry of Health in Brazil, which recommends pentavalent antimonials as the first drug of choice for the treatment of VL without complications, N-methylglucamine antimoniate was used [13].

#### Visceral leishmaniasis with HIV

Liposomal amphotericin B was employed as the drug of first choice, as recommended since 2015 by the Brazilian Ministry of Health guidelines. Before this date, antimoniate and amphotericin B were used to treat these patients [10].

#### HIV

All patients with a recent HIV diagnosis were treated with antiretroviral therapy (ART) according to the Brazilian Ministry of Health guidelines [15].

### Statistical analyses

Data were processed using SPSS software version 18.0 (IBM, Chicago, IL, USA). For descriptive statistics, the frequency, average, and standard deviation were calculated. The distributions of categorical variables were compared between groups using the Chi-squared test or Fisher’s exact test. For all analyses, a significance level of 5% was adopted.

### Ethics considerations

The research project was registered with Plataforma Brasil, and ethics committee approval was received from Maranhão’s University Hospital (HUUFMA; date of approval: September 28, 2013, report number: 409.351). The patients in the prospective study signed a free informed consent form, although the retrospective cases were released from this requirement by the ethics committee; nonetheless, their anonymity was assured from the time of data collection. Permission was also obtained from the Staging and Research Coordination of the State Department of Health of Maranhão.

## Results

In total, 252 patients were evaluated in this study, including 126 with VL/HIV coinfection and 126 with VL alone. The demographic and social characteristics of the study groups are provided in Table 1. Both groups primarily consisted of male patients. The mean age in the VL/HIV group was 36.9 ± 9.27 years (range, 19–68), compared to 32.8 ± 13.4 years (range, 18–85) in the VL group (*p* < 0.001). In both groups, patients were primarily native individuals from Maranhão’s countryside.

**Table 1:** Sociodemographic variables of the studied groups.

Variable	VL/HIV group (n = 126)		VL group (n = 126)		*P*
	*n*	*%*	*n*	*%*	
**Sex**					0.689
** Male**	113	89.7	110	87.3	
** Female**	13	10.3	16	12.7	
**Age group**					<0.001*
** 19–29 years**	19	23.0	70	55.6	
** 30–39 years**	49	38.9	28	22.2	
** 40–49 years**	38	30.2	11	8.7	
** 50+ years**	10	7.9	17	13.5	
**Place of residence**					0.153
** São Luís**	54	42.9	42	33.3	
** Other municipalities**	72	57.1	84	66.7	
**Occupation**					<0.001*
** Self-employed**	26	20.6	23	18.2	
** Agriculture**	25	19.9	59	46.8	
** Construction industry**	21	16.7	10	7.9	
** General services**	14	11.1	6	4.8	
** Housewife**	8	6.3	4	3.2	
** Other**	32	25.4	24	19.1	

VL, visceral leishmaniasis.

*Statistically significant difference between the groups, as determined using the Chi-squared or Fisher’s exact test (p < 0.05).

As shown in Table 2, the most frequent clinical complaints in the VL group were fever and anorexia (*p*<0.05). In the VL/HIV group, we found diarrhea, vomiting, bleeding and dyspnea (*p*<0.05). Physical examination data revealed that weight loss, fever, jaundice, hepatomegaly and splenomegaly were common in the VL group (*p*<0.05). In contrast, dyspnea and adenomegaly were detected in most cases in the VL/HIV group (*p =* 0.001). The frequency of skin/mucosal pallor was high in both groups but higher in the VL group (*p* = 0.001).

**Table 2:** Clinical and physical examination data for the two groups.

Variable	VL/HIV group (n = 126)		VL group (n = 126)		*p*
	*n*	*%*	*n*	*%*	
**Clinical complaint**					
** Fever**	66	52.4	115	91.3	<0.001*
** Hair loss**	20	15.9	15	11.0	0.466
** Anorexia**	97	77.0	112	88.9	0.019*
** Diarrhea**	66	52.4	32	25.4	<0.001*
** Vomiting**	37	29.4	21	16.7	0.024*
** Bleeding**	27	21.4	13	10.3	0.025*
** Cough**	38	30.2	38	30.2	1.000
** Dyspnea**	38	30.2	23	18.2	0.039*
**Physical symptom**					
** Alopecia**	11	8.7	4	3.2	0.107
** Weight loss**	66	52.4	103	81.7	<0.001*
** Fever**	22	17.5	75	59.5	<0.001*
** Jaundice**	22	17.5	46	36.5	0.001*
** Spotting**	11	8.7	1	0.8	0.005*
** Bleeding**	12	9.5	11	8.7	1.000
** Swelling**	15	11.9	10	7.9	0.399
** Dyspnea**	17	13.5	1	0.8	<0.001*
** Hepatomegaly**	89	70.6	105	83.3	0.024*
** Splenomegaly**	73	57.9	107	84.9	<0.001*
** Adenomegaly**	15	11.9	4	3.2	0.015*
** Abdominal protrusion**	46	36.5	17	13.5	0.052
**Skin/mucosa color**					<0.001*
** Normal color**	27	21.4	7	5.6	
** Pale**	99	78.6	119	94.4	

VL, visceral leishmaniasis.

*Statistically significant difference between the groups as determined using the Chi-squared or Fisher’s exact test (p < 0.05).

In general, visceral leishmaniasis manifested after HIV diagnosis, with an average interval of 2.6 years in 63% of the patients; in 37%, the diagnosis of VL occurred concomitant with that of HIV. In 100% of these patients, the diagnosis of VL/HIV was achieved by the observation of amastigotes in the bone marrow. Independent of the test used, serology for VL was only positive in 30% of coinfection cases by immunofluorescence (IFI) or the rapid test. In cases of VL without HIV, 100% of patients presented positive serology (IFI and/or the rapid test; Table 3).

**Table 3:** Diagnostic and laboratory data for the two groups.

Variables	VL/HIV group (*n* = 126)		VL group (*n* = 126)		*p*
	*n*	%	*n*	%	
**Diagnostic Test**					<0.001*
** Serological Test +**	38	30.2	100	100	
** Serological Test –**	88	69.8	0	0	
** Parasitological Test +**	126	100	62	49.2	
** Parasitological Test Ignored**	0	0	64	50.8	
**Red blood cells (million/mm**^**3**^**)**					0.216
** <4.6**	118	93.6	123	97.6	
** 4.6–6.2**	8	6.4	3	2.4	
**Hemoglobin (g/dL)**					0.748
** <14.0**	120	95.2	122	96.8	
** 14–17**	6	4.8	4	3.2	
**Hematocrit (%)**					0.065
** <40**	119	94.4	125	99.2	
** 40–54**	7	5.6	1	0.8	
**Leukocytes (1000/mm**^**3**^**)**					<0.001*
** <5000**	101	80.2	122	96.8	
** 5000–10,000**	22	17.5	4	3.2	
** >10,000**	3	2.3	0	0	
**Lymphocytes (%)**					<0.001*
** <20**	30	23.8	17	13.5	
** 20–40**	79	62.7	58	46.0	
** >40**	17	13.5	51	40.5	
**Platelets (1000/mm**^**3**^**)**					<0.001*
** <150,000**	79	62.7	107	84.9	
** 150,000–400,000**	45	35.7	17	13.5	
** >400,000**	2	1.6	2	1.6	
**Urea (mg/dl)**					0.729
** 10–50**	105	83.3	108	85.7	
** >50**	21	16.7	18	14.3	
**Creatinine (mg/dl)**					0.475
** ≤1.2**	110	87.3	105	83.3	
** >1.2**	16	12.7	21	16.7	
**AST (U/L)**					<0.001*
** 12–46**	76	60.3	32	25.4	
** >46**	50	39.7	94	74.6	
**ALT (U/L)**					<0.001*
** 3–50**	102	80.9	57	45.2	
** >50**	24	19.1	69	54.8	
**CD4 T-lymphocytes (cells/mm**^**3**^**)**					
** <200**	98	77.8	–	–	
** ≥200**	28	22.2	–	–	
**Viral load (copies/mL)**					
** <50**	19	15.1	–	–	
** 50–9999**	68	54.0	–	–	
** 10,000–100,000**	13	10.3	–	–	
** >100,000**	26	20.6	–	–	

VL, visceral leishmaniasis.

*Statistically significant difference between the groups, as determined using the Chi-squared or Fisher’s exact test (p < 0.05).

Table 3 shows the results of the comparative analysis of serum profiles between the groups at the time of admission for VL treatment. The VL group exhibited higher frequencies of leukopenia and thrombocytopenia than the VL/HIV group (*p* < 0.001). Abnormal AST and ALT levels were observed more commonly in the VL group (*p* < 0.001). Additionally, CD4 T-lymphocyte counts of fewer than 200 cells/mm^3^ and viral loads of 50–9999 copies/mL were observed in most coinfected patients.

The distribution of variables related to treatment is shown in Table 4. Patients who had been diagnosed with HIV prior to presenting with VL (63%) started ART before liposomal amphotericin B, and there was no interruption in treatment. In the remaining 37% who presented with VL at the time of HIV diagnosis, the treatment of leishmaniasis began before ART. The most commonly used medicines in the VL/HIV and VL groups were liposomal amphotericin B (Amb-L) and *N*-methyl meglumine antimoniate (Sb^v^), respectively (*p* < 0.001). The treatment duration for VL was no more than 10 days for most patients in the coinfection group (*p* < 0.001). In addition, 112 patients (88.9%) in the VL/HIV group remained hospitalized for more than a month, in most cases due to relapse, neurotoxoplasmosis complications, Kaposi sarcoma or ocular cytomegalovirus. None of the patients had tuberculosis or pneumocystosis concomitant with VL. Of particular importance, 11% of patients were hospitalized for other coinfections, without presenting any clinical or laboratorial manifestations of VL, but were diagnosed with VL during hospitalization after exhibiting cytopenia, with or without fever, and hepatosplenomegaly. Furthermore, relapse occurred in 36.5% of the coinfection group, and only one patient with VL without HIV relapsed (*p* < 0.001). Concerning ART, highly active antiretroviral therapy was administered to all patients, most commonly involving a nucleoside reverse transcriptase inhibitor in combination with a non-nucleotide reverse transcriptase inhibitor or protease, such as tenofovir + lamivudine in 113 (89.7%) patients, efavirenz in 54 patients (42.9%), and lopinavir/ritonavir in 52 patients (41.7%). Moreover, relapse occurred in 46 patients 36.5%) using ART, with 68% of the patients having CD4 levels ≤ 200 cells/μL and 44% with viral loads < 350 copies/mL. More deaths occurred in the VL/HIV group (11.1%) than in the VL group (2.4%).

**Table 4:** VL treatment in the two groups.

Variables	VL/HIV group (*n* = 126)		VL group (*n* = 126)		*p*
	*n*	%	*n*	%	
**VL treatment**					<0.001*
** Liposomal amphotericin B**	86	68.2	28	22.2	
** Amphotericin B**	24	19	8	6.3	
** *N*-methyl meglumine antimoniate**	8	6.4	87	69.1	
** More than one drug**	8	6.4	3	2.4	
**Duration of treatment**					<0.001*
** Up to 10 days**	91	72.8	9	6.4	
** 11–30 days**	27	21.6	59	46.8	
** More than 30 days**	7	5.6	59	46.8	
**Duration of hospitalization**					<0.001*
**None**	0	0	37	29.4	
** Less than 1 month**	14	11.1	84	66.6	
** 1–7 months**	109	86.5	5	4	
** More than 6 months**	3	2.4	0	0	
**VL relapse**					<0.001*
** Yes**	46	36.5	1	0.8	
** No**	80	63.5	123	99.2	
**Death**					0.009*
** Yes**	14	11,1	3	2.4	
** No**	112	88.9	123	97.6	

VL, visceral leishmaniasis.

*Statistically significant difference between the groups, as determined using the Chi-squared or Fisher’s exact test (p < 0.05).

## Discussion

The epidemiological characteristics of visceral leishmaniasis, an endemic disease in the northern and northeastern regions of Brazil, have changed, with the spread of HIV infection toward the rural areas and coincidence of the two diseases. In patients infected with HIV, VL can result in further immunosuppression and stimulate viral replication, leading to a more aggravated condition and greater risks of relapse and death [13, 10, 16, 17].

Although VL/HIV coinfection is variable among Brazilian regions, its incidence appears to be increasing across the country. According to Lindoso *et al*., the rates of coinfection were 0.7% in 2001 and 8.5% in 2012 [18]. In addition, the municipality of Rondonopolis, Mato Grosso, reported 81 autochthonous cases of coinfection between 2011 and 2016; when combined with 10 other municipalities, these areas were responsible for 15% of the cases of VL in Brazil, with a coinfection rate of 9.9% [19]. Overall, coinfection rates have increased, with data from SINAN-NET indicating significant increases, reaching 9.08% in 2017. [19,10].

Data were collected for 126 patients with VL/HIV coinfection at the referral hospital where the study was performed between 2007 and June 2017. This cohort represented nearly 50% of all reported cases in the state of Maranhão through December 2017 [20]. The finding of a higher incidence of VL in males with VL alone and in those coinfected with HIV is supported by other findings, including epidemiological data from the Health Ministry, which reported that male patients accounted for 64.8% and 91.6% of all cases of VL and VL/HIV, respectively [13,21,22]. The report of a higher prevalence in males is found in several articles in Brazil, both in patients with VL and in those with coinfections. Although definitive explanations of the reason for higher incidence rates in men are lacking, some authors report that this may be associated with greater exposure, factors related to sex or factors related to genetic modulation [23,24,25]. Similarly, the finding that the mean age was lower in the VL group agrees with data reported by Hurissa *et al*., who found a mean age of 23.5 years among patients with VL alone. However, Cota *et al*. reported no significant difference in age between VL/HIV and VL groups in a study of 90 patients [22,26].

The majority of diagnosed patients resided in Maranhão’s countryside, as corroborated by Furtado, who between 2000 and 2009 evaluated the residences of patients with VL in the state of Maranhão based on the National System for Reporting Harms by applying a method for risk identification. According to this analysis, incidence rates were higher in the cities of Caxias, Imperatriz, Presidente Dutra, Codó, and Barra do Corda, also highlighting the appearance of cases in countryside cities in which no VL case had been previously reported [27]. By observing the spread of VL to the interior of the state, it is possible to perceive the juxtaposition that occurs between VL and HIV infections in people living in an area that provides both pathogens an adequate environment in which to develop [28].

In line with our findings, Cota *et al*. described fever and hepatosplenomegaly as the most frequent complications in patients with VL alone[26]. In contrast, Hurissa *et al*. found typical VL complications in both groups [22]. In their study, fever was observed in 91.3% of patients with VL alone compared to 52.4% of patients with VL/HIV coinfection; however, this symptom was present in 100% of patients diagnosed in the state of Mato Grosso do Sul between 2000 and 2006 and 92% of patients diagnosed in the state of Ceará in 2015 [21,29]. Many Brazilian studies have reported the presence of splenomegaly followed by fever, weight loss, and asthenia. Nonetheless, in 81 patients with VL/HIV coinfection in Ceará [30] and in another study that identified weight loss, weakness, fever, and hepatosplenomegaly as the most common physical changes in 65 patients with VL/HIV coinfection [31], the most common symptoms were skin pallor, hepatomegaly, and splenomegaly, followed by weight loss and fever. These findings emphasize a low proportion of patients with splenomegaly (57.9%) when compared to patients with VL without HIV (84.9%), which is different from what was observed by Sousa-Gomes *et al*. (79.7%) [32] but similar to what was reported by Pintado *et al*. [33]. In a systematic review of the epidemiological, clinical, and laboratory aspects of VL associated with HIV, the 10 most commonly reported clinical manifestations among 15 studies were skin pallor, splenomegaly, fever, weight loss, hepatomegaly, cough, diarrhea, bleeding, asthenia, and jaundice [34]. In addition, a review by Lindoso *et al*. reported that although the clinical manifestations of coinfected patients are similar to those without HIV, weakness, cough, diarrhea, malnutrition and weight loss were found in a higher proportion in the HIV group than in the non-HIV group [35].

Laboratory diagnosis is still considered a challenge for VL, especially when VL is associated with HIV. In this study, patients without HIV presented positive serology for VL, regardless of the methodology used (IFI or the rapid test), yet it should be considered that in an area of high endemicity, despite the classic clinical manifestations in these cases, some cases may be misdiagnosed owing to a lack of positive serology. Because the positivity rate was 30.2% among the cases of coinfection, this is not an effective method for diagnosis. In contrast, parasitological examination revealed amastigote forms in all cases of coinfection and in all cases of VL without HIV. In a review carried out by Lindoso *et al*. [18], the effectiveness of diagnosis by the rapid test and IFI showed low sensitivity in 113 VL/HIV-coinfected patients. The same author, in 2018, reported rapid test sensitivity ranging from 46.6% to 81% [35]. Other important research showing higher positivity for parasitological diagnosis and lower positivity for immunological diagnosis was performed by Sousa-Gomes *et al*. [32], even though the positivity rate of serological diagnosis was higher than that observed in the present study. More than half of the cases in this study were from cities in the interior of Maranhão, where there is little infrastructure to support diagnosis by direct parasitological examination, which is one of the causes of late diagnosis.

Pancytopenia was observed in both studied groups, in contrast to the findings of Cota *et al*. and Hurissa *et al*., who reported more pronounced thrombocytopenia in immunocompetent patients [25,26]. Pancytopenia related to HIV can be multifactorial due to the direct effects of the virus, ineffectiveness of hematopoiesis, existence of an infiltrating disease in the bone marrow, nutritional deficiencies, peripheral destruction, and toxic medication effects [36]. In contrast, hematological alterations caused by VL are well known, as amastigotes proliferate in the mononuclear phagocytic system, mainly in the spleen, liver, and bone marrow, resulting in phagocytic organ disorders and hematological alterations [37]. Pancytopenia was observed in both groups studied, with most patients with VL infection alone, unlike in Tavora *et al*., in which most cases of pancytopenia were observed in the VL/HIV coinfection group [29]. Increased creatinine levels were observed in less than 20% of the cases in both groups, which was similar to the results of the study by Mahajan *et al*., in which a 16.5% increase in the creatinine levels was observed in 97 patients with VL/HIV coinfection [35%] [38]. By analyzing 49 patients with VL/HIV coinfection, Sinha *et al*. found a mean creatinine level of 0.9 mg/mL [39]. The present study shows that the VL group without coinfection had higher rates of increase in transaminases, as additionally reported by Lima *et al* and De Souza *et al* [40, 41].

CD4 T-lymphocytes have been quantified by several authors who emphasized, similar to our study, the presence of immunosuppression, with counts lower than 200 cells/mm^3^ in a large portion of coinfected patients at the time of VL diagnosis [21,39,40,42,43]. However, we should highlight that HIV was diagnosed first in 64.3% of patients with VL/HIV coinfection; accordingly, these patients were in a more immunologically suppressed state due to VL. Regardless, most patients had viral loads of more than 50 copies; although most patients were receiving ART, a lack of therapeutic adoption or virological failure is suspected in this group. Despite previous studies describing that the use of ART improves the survival of patients with VL, recurrences continue to be reported in cases of coinfection. Another important point is that ART does not benefit patients exposed to the two pathologies outside the urban area, as occurred for more than half of the coinfected patients in this study, who have difficulty accessing the health system for diagnosis [33, 32].

The manifestation of VL as immune reconstitution has already been proposed, albeit in a limited number of studies [44]. Nonetheless, 11% of our patients had VL diagnosed during hospitalization for the treatment of other opportunistic diseases while receiving ART.

Treatment for VL was administered according to the Health Ministry recommendations, with AmB-L being the drug of choice for patients with VL/HIV coinfection starting in 2013; Sb^v^ was used to treat patients with non-severe VL [12]. Brazilian authors have reported treatments that reflect guidance from the Health Ministry, drawing attention to the adverse effects of AmB, including kidney toxicity and reactions during infusion. Toxicities linked to Sb^v^ administration have also been reported, in some cases requiring substitution with AmB, and some reports cite the occurrence of pancreatitis and cardiotoxicity [21,26,41,45]. The treatment duration for VL varies according to the therapeutic response using the criteria adopted by the Ministry of Health of Brazil, which call for a minimum duration of 10 days [13]. However, we observed therapeutic failure, even after increasing the duration of AmB-L administration, rendering supplemental therapy with Sb^v^ necessary (data not shown). AmB-L has been considered promising for the treatment of VL/HIV coinfection since 1999, when it was approved by the FDA [46]. Although AmB-L is associated with fewer side effects than AmB, VL/HIV coinfection remains associated with high rates of recurrence, as noted in the present study. Lindoso *et al*. highlighted that no standard therapeutic regimens have been developed for VL in the United States [18]. Additionally, Cota *et al*. reported recurrence and mortality rates of 37% and 8.7%, respectively, in a group of 46 patients with VL/HIV coinfection compared to 2.5 and 4.7%, respectively, in 44 patients with VL without HIV[26]. In a sample of 128 patients with VL, Oliveira reported recurrence and death rates of 7.8 and 4.7%, respectively [47]. These results differ from those in the present study, in which recurrence occurred in 0.8% and death was observed in 2.4% of patients with VL monoinfection, but between the group VL/HIV coinfection we observed a recurrence and mortality rates of 36,5% and 11.1%, respectively, like Cota *et al*.[26].

This study had several limitations. Because of the use of secondary data, mainly in the retrospective analysis, incomplete data were obtained for some examinations. Another limitation was the the difficult in rescuing patients with VL without HIV. They were hospitalized for a short time, returning to their cities, only having news in case of relapse, as they returned to hospital. The patients with VL/HIV coinfection in this study represented almost half of the 331 cases of coinfection in the state of Maranhão over the period of 2007–2017, and we only included patients for whom complete data were available, increasing the statistical power of the analyses.

In summary, our study found that VL is a public health issue in northeastern Brazil, especially in Maranhão, where its incidence is increasing. The severity of the disease is exacerbated by coinfection with HIV, which modifies the epidemiology and clinical presentation of VL. Thus, the classic triad of fever, pallor, and hepatosplenomegaly should not be expected. We also concluded that treatment remains challenging, despite advances in ART. Overall, a definitive response to treatment for VL cannot be predicted when the disease is associated with HIV.

## Supporting information

S1 Checklist STROBE checklist. (DOC)

S1 Table Descriptive analyses of sérum data. (PDF)
