# Supplementary material for: Visceral leishmaniasis in Northeast Brazil: What is the impact of HIV on this protozoan infection?
Source: PLoS One. 2019 Dec 5;14(12):e0225875. doi: 10.1371/journal.pone.0225875 (PMC6894860; doi:10.1371/journal.pone.0225875)
Supplement: S1 Table — (PDF) [file pone.0225875.s002.pdf]

**S2. Descriptive analysis of serum data.**

| Variáveis | VL/HIV group (*n* = 126) | | | | VL group (*n* = 126) | | | |
|---|---|---|---|---|---|---|---|---|
| | mean | ±sd | median | 1ºQ-3ºQ | mean | ±sd | median | 1ºQ-3ºQ |
| Red blood cells (million/mm$^3$) | 3.3 | ±0.8 | 3.4 | 2.8-3.8 | 3.2 | ±0.7 | 3.3 | 2.7-3.6 |
| Hemoglobin (g/dL) | 10.5 | ±12.8 | 9.2 | 7.9-10.9 | 9.4 | ±7.1 | 8.4 | 7.3-9.9 |
| Hematocrit (%) | 28.5 | ±7.8 | 28.2 | 24.5-33.9 | 25.5 | ±6.5 | 25.4 | 21.7-29.6 |
| Leukocytes (1000/mm$^3$) | 3.3 | ±2.5 | 2.5 | 1.6-4.6 | 2.1 | ±1.4 | 1.7 | 1.4-2.7 |
| Lymphocytes (%) | 27.2 | ±13.3 | 27.6 | 18.4-36.6 | 37.2 | ±16.1 | 35.1 | 26.0-46.8 |
| Platelets (1000/mm$^3$) | 154.5 | ±99.2 | 132.0 | 92.4-187.2 | 96.5 | ±74.4 | 83.4 | 50.0-121.0 |
| Urea (mg/dl) | 35.5 | ±15.1 | 34.0 | 24.0-47.0 | 34.4 | ±21.8 | 29.0 | 22.4-40.0 |
| Creatinine (mg/dl) | 0.9 | ±0.7 | 0.8 | 0.6-1.1 | 0.9 | ±0.5 | 0.8 | 0.7-1.0 |
| AST (U/l) | 55.5 | ±49.5 | 42.0 | 27.0-59.3 | 145.6 | ±220.3 | 85.5 | 45.0-135.0 |
| ALT (U/l) | 40.9 | ±36.0 | 29.0 | 20.0-46.0 | 106.9 | ±176.4 | 59.5 | 32.0-105.0 |
| CD4 T-lymphocytes (cells/mm$^3$) | 145.2 | ±126.8 | 120.5 | 54.0-186.3 | - | - | - | - |
| Viral load (1000 copies/mL) | 115.90 | ±516.72 | 0.25 | 0.05-35.23 | - | - | - | - |

+±sd = standard deviation. 1ºQ = first quartile. 3ºQ = third quartile. AST = Aspartate Aminotransferase. ALT = Alanine Aminotransferase.
